# Chronomodulated Drug Delivery System of Salbutamol Sulphate for the Treatment of Nocturnal Asthma

**DOI:** 10.4103/0250-474X.43000

**Published:** 2008

**Authors:** J. Qureshi, Mohd. Amir, Alka Ahuja, Sanjula Baboota, J. Ali

**Affiliations:** Department of Pharmaceutics, Faculty of Pharmacy, Jamia Hamdard, (Hamdard University), New Delhi-110 062, India

**Keywords:** Nocturnal asthma, time-dependent drug delivery system, circadian rhythm, swelling layer, rupturing layer, salbutamol sulphate

## Abstract

A time dependent pulsed release system consisting of an effervescent core surrounded by consecutive layers of swelling and rupturable polymers was prepared and evaluated. The cores containing salbutamol sulphate as bioactive agent were prepared by direct compression method using different ratios of microcrystalline cellulose and effervescent agent and then coated sequentially with an inner swelling layer containing a hydrocolloid, hydroxypropylmethylcellulose E5 and an outer rupturable layer having Eudragit RL/RS (1:1). The effects of various processing and formulative parameters on the performance of system were studied. The rupture and dissolution tests were studied using the USP paddle method at 50 rpm in 0.1 N HCl and phosphate buffer pH 6.8. The lag time of the drug release decreased by increasing the inner swelling layer and increased by increasing the rupturing layer level. All the results obtained in the present study suggest that osmotic pumping effect was involved which eventually lead to the drug release.

Nocturnal asthma, a condition prevalent in two-thirds of the asthmatics, is defined as a variable night time exacerbation of the underlying asthma condition associated with increase in symptoms and need for medication, increased airway responsiveness and worsening of lung function. Symptoms typically occur between midnight and 8 am, especially around 4.00 am^1^–^3^. It is inconvenient to take the medication at midnight. The maintenance of constant drug level is not always desirable for the optimal therapy. A drug should be delivered only when and/or where it is needed at the minimum required dose^4^. For the drugs to follow circadian rhythm, like in asthma, a reasonable and an acceptable rationale is a delivery system capable of releasing drugs in a pulsatile fashion rather than as a continuous delivery at the predetermined time/site following oral administration^5^,^6^.

Thus, this study attempts to design and evaluate a chronomodulated drug delivery system of salbutamol sulphate, a selective β_2_ receptor blocker for the treatment of nocturnal asthma. It was aimed to have a lag time of six hours i.e., the system is taken at the bed time and expected to release the drug after a period of 6 h i.e., at 4.00 am when the asthma attacks are more prevalent. Such time-controlled pulsatile delivery can be achieved mainly with drug containing cores, which are covered with release controlling layers. The core serves as reservoir, and the release controlling layers protect the core from the environment e.g., water, acidic pH and enzymes until the drug is released after a predetermined lag phase. The coatings can erode/dissolve, rupture or alter their permeability at the required time. Single unit rupturable pulsatile drug delivery system was chosen as the model system over erodible pulsatile drug delivery system or Pulsincap^®^ and PORT^®^ systems^7^ because of ease of manufacturing, better reproducibility of the lag time and rapid drug release after a lag time. The proposed system consisted of core tablet coated with an inner swelling layer (HPMC E5) and an outer rupturable coating (Eudragit RL/RS) aimed to release the drug after a lag time of 6 h.

## MATERIALS AND METHODS

Salbutamol sulphate was obtained as a gift sample from Kee Pharma Ltd. (India). HPMC E5 and Eudragit RL/RS were obtained from Ranbaxy research labs (Gurgaon, India). PEG 4000 was purchased from CDH, Mumbai. Sodium chloride and sodium bicarbonate were purchased from Merck, Mumbai. All other chemicals used were of analytical reagent grade.

### Preparation of core tablets:

The core tablets containing salbutamol (4 mg/tablet) were prepared using the following composition: 2% w/w salbutamol sulphate, 30% w/w sodium chloride, 20% w/w microcrystalline cellulose (MCC), 20% w/w Starch, 20% w/w sodium bi carbonate, 6% w/w lactose 1% w/w magnesium stearate and 1% w/w talc. All excipients were mixed for 20 min and passed through a 125 mesh size sieve and directly compressed in to 200 mg tablets using 8 mm round concave punches on a rotary tablet machine using a force of 3000 kg. Tablet hardness was measured with Monsanto hardness tester and was found to be 3.0±0.5 kg. Core tablets without sodium chloride and sodium bicarbonate were also prepared and these ingredients were replaced with lactose as inert filler.

### Development of pulsatile release tablets:

Pulsatile release tablets were prepared by coating core tablets with an inner swelling layer comprising of HPMC E5 (20% w/w of core tablet) and an outer polymeric layer consisting of Eudragit RL/RS (1:1, 6 mg/cm^2^) dispersed in water/ethanol solution (60/40 v/v) using PEG 4000 (5% w/w of polymer content) as a plasticizer. Inner swelling layer was applied by direct compression whereas outer polymeric layer was incorporated by conventional pan coating. The process conditions used were as: inlet temperature 40-45°; product temperature 33-35°; pan speed 30 rpm; spray rate 4-6 g/min; atomizing pressure 1.2 bar. The coated tablets were further dried in a coating pan for 15 min at 35°. After finishing the coating process the tablets were then placed in an oven at 50° for 2 h to remove the solvent. The surface morphology of the tablet was smooth and uniform in appearance.

### *In vitro * drug release studies:

The *in vitro* drug release from coated tablets was carried out using USP paddle apparatus at 50 rpm and 37±0.5°. HCl (0.1 N) and phosphate buffer (pH 6.8) were used as the dissolution medium. Initially tablets were subjected to dissolution in 0.1 N HCl for 2 h and after that media were changed to phosphate buffer (pH 6.8). The samples were withdrawn at regular intervals and analyzed by UV spectrophometer (Shimadzu UV/Vis 1601) at 276 nm for the presence of the drug. Dissolution tests were performed in triplicate.

### Rupture test:

The time at which the outer coating layer starts to rupture is defined as the lag time. It was determined visually by using the USP dissolution apparatus II (900 ml of 0.1 N HCl for initial 2 h and then media was changed to phosphate buffer pH 6.8, 37±0.5°, 50 rpm, (n=3)^8^.

### Effect of outer polymer concentration and water uptake performance:

To study the effect of outer polymeric layer concentration on lag time, core tablets were coated with different levels of Eudragit RL/ RS (1:1) i.e. 4, 6, 8 and 10% w/w (inner swelling layer remained the same). The % water uptake capacity of tablets was determined in the containers filled with 100 ml of 0.1 N HCl placed in a biological shaker at 37°. Speed of shaker was adjusted to 75 rpm. Tablets were removed from containers at predetermined regular intervals, blotted with tissue paper, weighed and again placed in medium till the outer coating of tablet started to rupture. The % water uptake was calculated using the formula, % Water uptake = ((W_t_-Wo)/Wo)×100, where W_t_ is weight of wet tablet at time t and Wo is weight of dry tablet^9^.

### Effect of inner swelling layer on the lag time:

Core tablets were coated with 10% w/w, 20% w/w and 30% w/w of HPMC E5 as inner swelling layer and subjected to dissolution study as described in method 2.2. Outer polymer layer remained the same (Eudragit RL: RS, 1:1). Effect of swelling layer concentration over lag time and release behavior was observed using a spectrophotometer as described in method under *in vitro* drug release studies.

### Effect of sodium bicarbonate and osmotic agent (sodium chloride) on release characteristics:

Core tablets with and without sodium bicarbonate and sodium chloride were prepared individually and coated with the same polymeric inner (HPMC E5) and outer layers (eudragit RL:RS 1:1). To observe the effect of osmotic agent over lag time, the tablets were subjected to *in vitro* dissolution study and release profiles were compared with the formulations containing an osmotic agent and sodium bicarbonate in their inner core.

### Effect of paddle speed on the lag time and release characteristics:

Coated tablets were subjected to *in vitro* dissolution study at different paddle speeds (50 and 100 rpm). Other conditions remained the same as described. Effect of paddle speed on release behavior and lag time was observed and analyzed using a spectrophotometer^10^.

### Scanning electron micrography:

The tablet was transversely cut and was sputtered with gold (30 mm thick) under vacuum and microscopy was performed with a LEO-435 VP, PC based digital scanning electron microscope. It was viewed for the presence of polymer layer at 600X magnification.

### Stability studies:

The shelf life was determined using accelerated stability studies as per WHO guidelines. Optimized tablets in glass containers were kept at temperatures of 40±0.5°, 50±0.5° and 60±0.5° at 65% RH for 90 d. Samples were withdrawn at regular intervals and samples were analyzed using HPLC with a C_18_ column. The mobile phase consisted of acetonitrile:water:glacial acetic acid: triethylamine (55:45:0.1:0.1) at a flow rate was adjusted to 1.5 ml/min and the samples were analyzed at 276 nm^11^. The log percentage drug remaining was plotted against time and slope was determined. The effect of temperature on the degradation was studied by plotting log K vs. 1/T. The value of K at 25° was extrapolated from the plot and shelf life was calculated by substituting K_25_ in the equation, T_0.9_ = 0.1054/K_25_.

## RESULTS AND DISCUSSION

**Fig. 1:**
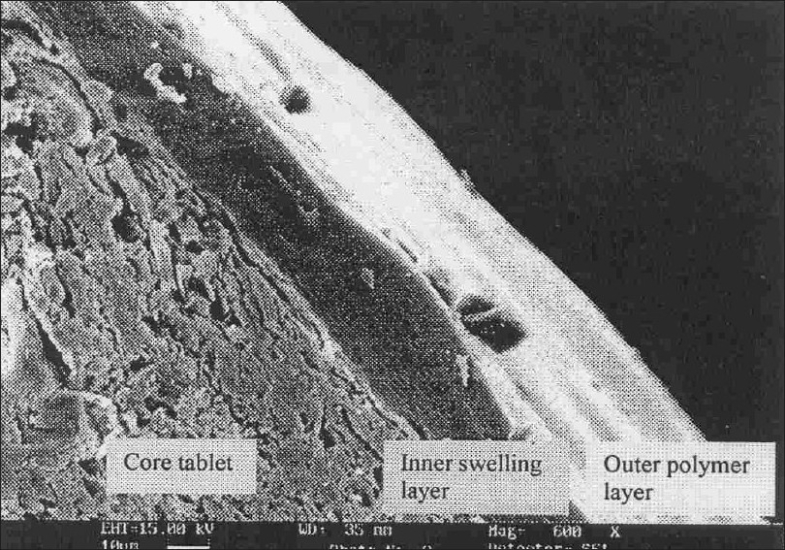
Scanning electron micrograph of the tablet showing core tablet with different layers of polymers.

**Fig. 2:**
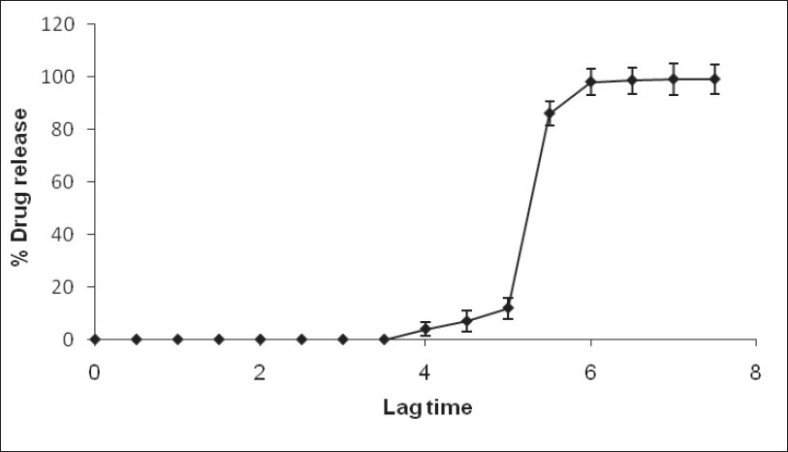
*In vitro* dissolution behavior of developed pulsatile drug delivery system.

The rupturable pulsatile drug delivery system consisted of a core, a drug containing reservoir, inner or intermediate swelling layer and an outer water insoluble but permeable coating. The swelling layer consisted of hydroxypropyl methyl cellulose (HPMC E5) it was chosen because of its swelling nature and its eroding behavior and was applied by direct compression method. The rupturable coating consisted of a plasticized mixture of Eudragit RL/RS (1:1), as it formed a mechanically weak and semipermeable film, which could rupture easily upon exposure to the dissolution media and was water insoluble, swellable and pH independent film former. As the numbers of hydrophilic quaternary ammonium groups present in Eudragit RL were much higher as compared to RS, faster drug release was observed from it. The drug release could be modified by adjusting the ratios of these two polymers in combination. fig. 1 shows a transverse-sectioned photograph of time-dependent tablet. The parts of the tablet as seen in the figure, the core (MCC, sodium chloride, sodium bicarbonate and drug), an intermediate swelling/eroding layer comprising of HPMC E5, and homogenous rupturable layer consisting of Eudragit RL/RS mixture (1:1). Water influx was through the semipermeable rupturable outer coating which leads to the expansion and erosion of an intermediate layer, which ultimately resulted in rupture of the outer coating. The drug was then released within a short time after a definite lag time period. The dissolution studies were carried out to investigate the release behavior of the developed system. Salbutamol sulphate is a freely water soluble drug. There was no drug release prior to the breaking of outer coating (fig. 2).

**Fig. 3:**
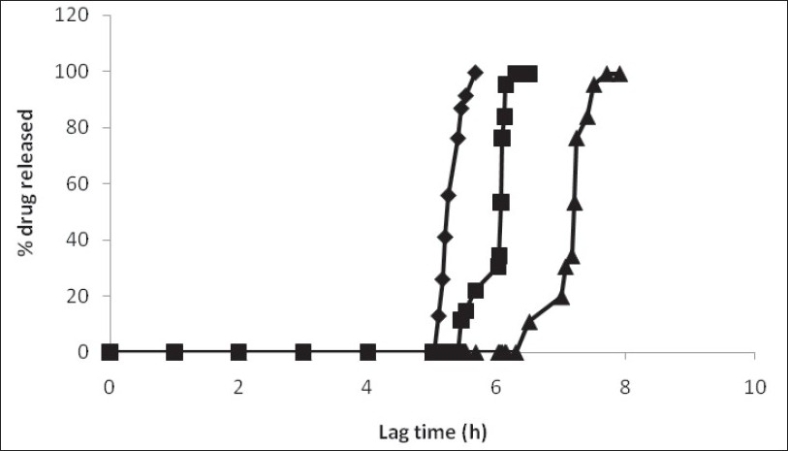
Effect of inner swelling layer concentration on the lag time of the time-dependent release tablet. Inner swelling layer concentrations were, 30% w/w (–♦–), 20% w/w (–■–) and 10 % w/w (–▲–).

Thickness of the swelling layer was the critical parameter which influenced the rupture of outer coating. The lag time of tablet decreased with increasing level of swelling layer. As the amount of swelling agent (HPMC E5) increased, it exerted more pressure over the outer layer resulting in rapid rupturing of the tablet. The expanded swelling layer facilitated the entry of dissolution medium to the core containing an effervescent agent, which further synergized the rupturing of the outer layer. After breaking of the outer layer the drug release from the time dependent release tablet with 10% w/w HPMC E5 layer was lower compared to that from the tablet with 20% w/w HPMC E5 layer. Increase in inner swelling layer concentration (30% w/w) resulted in early burst of tablet. A 10% HPMC E5 layer might not be enough for the complete rupture of the outer layer whereas a 20% w/w concentration of inner swelling layer was found to be optimum to rupture the outer polymeric layer (fig. 3).

**Fig. 4:**
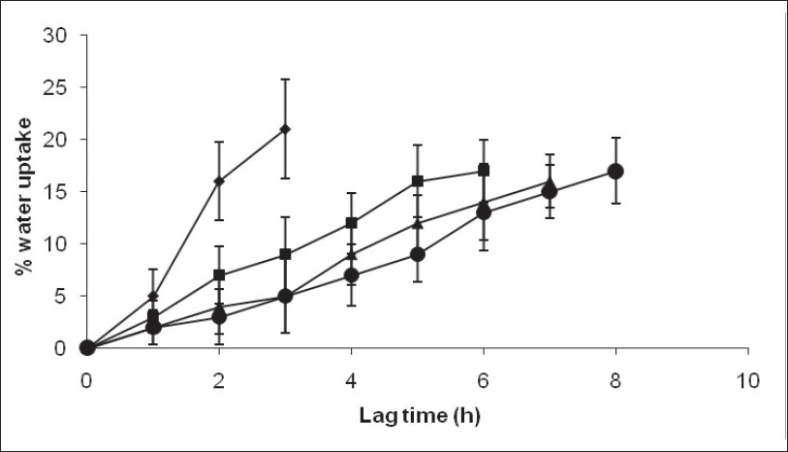
Effect of outer coating on % water uptake and lag time of developed pulsed release tablet. Eudragit outer coating levels used were 4 mg/cm^2^ (–♦–), 6 mg/cm^2^ (–■–), 8 mg/cm^2^ (–▲–) and 10 mg/cm^2^ (–●–).

The water uptake capacity and drug release before the rupture of tablet was dependent on outer acrylate polymer coating. The lag time increased with increased outer coating level. A fast and complete release was observed at 4 and 6 mg/cm^2^ Eudragit coating level. With higher coating level (8 and 10 mg/cm^2^) of Eudragit, a slower release was observed after lag time, due to lower degree of rupturing (fig. 4). Increased outer coating level decreased the water uptake. This could be explained by the greater degree of mechanical strength of the thick coating.

**Fig. 5:**
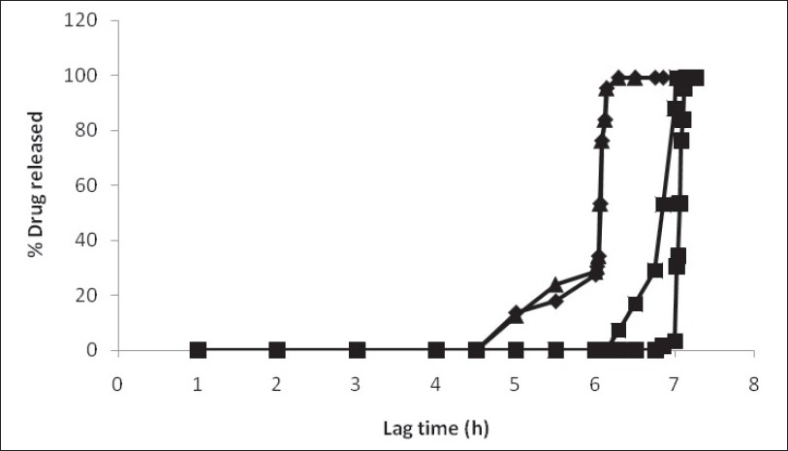
Effect of sodium chloride and sodium bicarbonate on the lag time of the pulsed release tablet. With sodium chloride (–♦.–) and without (–■–). With sodium bicarbonate (–▲–) and with out (–■–).

To generate osmotic pressure inside the core, sodium chloride was added as an osmotic agent. In order to investigate the effect of osmotic pressure, tablets with and without the osmotic agent (NaCl) were prepared and coated with the 20% w/w HPMC E5 as swelling layer and 6 mg/cm^2^ of Eudragit RL/RS (1:1) as an outer rupturable layer. These tablets were subjected to dissolution studies and their release profile was compared. The presence of an osmotic agent helped in drawing water towards the tablet which resulted in shortening of lag time^12^. Similarly, the effect of sodium bicarbonate on lag time was observed by conducting the dissolution study for the formulations developed with and without sodium bicarbonate. The tablets with sodium bicarbonate in their core showed slightly lower lag time due to the generation of carbon dioxide, which resulted in building up of pressure inside the core and helped in early rupturing of the outer polymeric layer^13^ (fig. 5). From these observations, it can be assumed that the first step in drug release is penetration of water in the core tablet by diffusion through Eudragit film and the rate and amount of water entered is dependent on outer film thickness. The HPMC E5 layer was swelled when it came in contact with water. No remarkable increase in volume was observed before water reached the HPMC layer. Initially a short period of no expansion was observed, after that fast volume expansion was observed. The penetration rate of water accelerated due to polymer chain relaxation. When the water reached the core, sodium chloride and drug dissolved and an osmotic gradient across the membrane was produced. Further, osmotic pressure played a key role to imbibe water from the environment continuously. Meanwhile, the films stretched faster as soon as the osmotic pressure gradient developed. After an optimum pressure development fractures were stretched into the film leading to faster release of the drug. At this stage the membrane was converted from semi permeable membrane to porous membrane^14^.

**Fig. 6:**
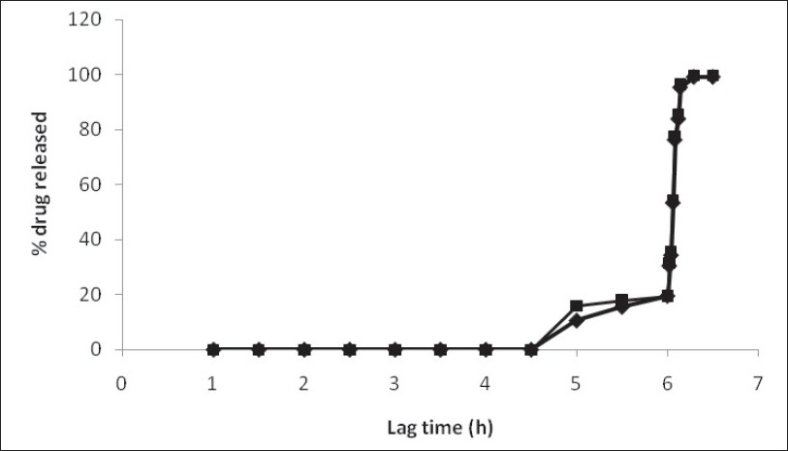
Effect of paddle rotation (rpm) of dissolution apparatus on the lag time and release profile of pulsed release tablet. Paddle rotation 50 rpm (–♦–) and 100 rpm (–■–).

**Fig. 7:**
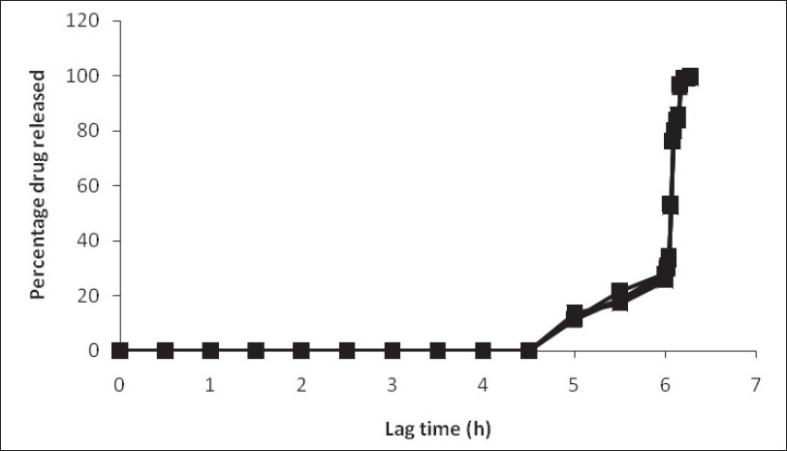
Effect of pH of dissolution medium on lag time of pulsed release system. pH 1.2 (–♦–), pH 5.5 (–■–), pH 6.8 (–▲–) and pH 7.2 (–■–).

Drug release behavior under different rotational speeds (50 and 100 rpm) and in different pH values was determined as shown in figs. 6 and 7. When such timed release tablets are orally administered, they are transferred from stomach to small intestine or colon while retaining the intact tablet shape. During this transit process, the pH of gastrointestinal juice around the tablet changed markedly from pH 1.2 to pH 8.0^15^. Therefore determination of dissolution behavior in various dissolution media was necessary. The dissolution of drug from swelling layered tablet (dry coated) was determined in pH 1.2, pH 5.5, pH 6.8 and pH 7.2 buffer solutions^16^. No significant difference in drug release was observed for release study in different pH or under different rotational speeds. This shows an advantage for the system, as it predicts no change in the performance of the system at increased gastric motility.

Scanning electron micrograph revealed that the coated tablet had a fairly smooth and tight surface. It also revealed the presence of core, inner swelling layer and outer polymeric layer (figs. 1 and 2). The coating layers were satisfactorily uniform.

Accelerated stability studies indicated a shelf life of 1.9 y. The formulation was fairly stable as revealed by stability studies conducted as per WHO guidelines. The degradation rate constant (K) was found to be 6.139×10^-5^ and degradation of the drug was 0.76% over a period of 3 months.

A chronomodulated drug delivery system for salbutamol sulphate for the treatment of nocturnal asthma was successfully developed. The system was found to be satisfactory in terms of release of the drug after a lag time of 6 h. The dosage form can be taken at bed time and will release the contents in the early hours of morning when the asthmatic attacks are more prevalent. The release of the drug was sharp and complete after the lag time, which is necessary for any pulsatile drug delivery system.
